# G2PMineR: A Genome to Phenome Literature Review Approach

**DOI:** 10.3390/genes12020293

**Published:** 2021-02-20

**Authors:** John M. A. Wojahn, Stephanie J. Galla, Anthony E. Melton, Sven Buerki

**Affiliations:** Department of Biological Sciences, Boise State University, Boise, ID 83725, USA; stephaniegalla@boisestate.edu (S.J.G.); anthonymelton@boisestate.edu (A.E.M.); svenbuerki@boisestate.edu (S.B.)

**Keywords:** genotype, phenotype, G2P, literature review, literature mining

## Abstract

There is a gap in the conceptual framework linking genes to phenotypes (G2P) for non-model organisms, as most non-model organisms do not yet have genomic resources readily available. To address this, researchers often perform literature reviews to understand G2P linkages by curating a list of likely gene candidates, hinging upon other studies already conducted in closely related systems. Sifting through hundreds to thousands of articles is a cumbersome task that slows down the scientific process and may introduce bias into a study. To fill this gap, we created G2PMineR, a free and open source literature mining tool developed specifically for G2P research. This R package uses automation to make the G2P review process efficient and unbiased, while also generating hypothesized associations between genes and phenotypes within a taxonomical framework. We applied the package to a literature review for drought-tolerance in plants. The analysis provides biologically meaningful results within the known framework of drought tolerance in plants. Overall, the package is useful for conducting literature reviews for genome to phenome projects, and also has broad appeal to scientists investigating a wide range of study systems as it can conduct analyses under the auspices of three different kingdoms (Plantae, Animalia, and Fungi).

## 1. Introduction

The post-genomics era is an exciting time to conduct research. Lower-cost sequencing technologies, candidate gene approaches, and genome-wide association studies (GWAS) allow researchers to unravel the genomic basis of traits (i.e., genome to phenome research, or G2P) across many organisms, which allows researchers to predict the responses of organisms to a changing global landscape [1,2,3,4,5,6]. G2P studies have a wide range of applications, such as studying cancer in humans [7], phenotypic variation in wild flora and fauna [4,8], or genes associated to pathogenicity in disease-causing organisms [9]. While the number of G2P studies in non-model organisms is increasing, there are challenges associated with starting G2P research from the ‘ground-up’, as genomic resources are often not readily available for non-model organisms [10,11,12]. This gap in the conceptual framework limits our ability to address how non-model organisms are responding to a rapidly changing world [12].

To address this gap, researchers often perform GWAS or outlier analyses using high-throughput sequencing outputs to associate phenotypes of interest with a list of candidate genes, with the validation of these genes afterwards through a thorough literature search [13,14]. While searching for genes is a popular choice for most researchers with relatively few genomic resources available, others use a forward genetic approach by mining genomes for known genes of interest, hinging upon other studies already conducted in closely related systems [15]. For both approaches, a thorough literature review is essential for understanding genes underpinning traits of interest within a taxonomic framework [15]. However, in the post-genomics era, with hundreds of thousands of articles to consider, this is a cumbersome task, which makes bridging the G2P gap difficult [15]. Furthermore, manual curation of genes of interest can introduce bias into a study [16]. Automated text mining soft offers a solution to this gap, however to our knowledge most available programs are focused primarily on the social sciences (e.g., sentiments analyses [17]) or scientometrics (e.g., bibliometrix [18]) and these do not work well within the G2P framework.

In this paper, we describe G2PMineR, a free and open-source R-package [19] literature mining tool that we developed specifically for G2P research. Using automation, G2PMiner makes the G2P review process of tens of thousands of studies (based on their abstracts, which are easily retrieved and often the only freely available part of a study) efficient and unbiased and presents hypothesized associations between genes and phenotypes within a taxonomical framework. This allows the user to make testable G2P hypotheses more quickly. Given that our research group studies plant genomics, we originally developed G2PMineR using manually vetted plant reference datasets. To expand the applicability of this tool, we also included pre-compiled datasets for animals and fungi. We anticipate that these datasets can be refined through feedback from the scientific community as this tool is disseminated and used in diverse applications (see Section 2.4 for more details).

## 2. Materials and Methods

The methods presented here are an abbreviated version. To see a full description of each function in the package and its place in the pipeline, please see the G2PMineR GitHub page at https://buerkilabteam.github.io/G2PMineR_Web/ (accessed on 2 February 2021). G2PMineR can be downloaded from GitHub at BuerkiLab/G2PmineR using the install_github function from the R package devtools [20]. For each function implemented in the package and referred to in the text: objects and arguments are underlined and column names in a data frame are in “quotation”.

### 2.1. General Structure

**Figure 1:**
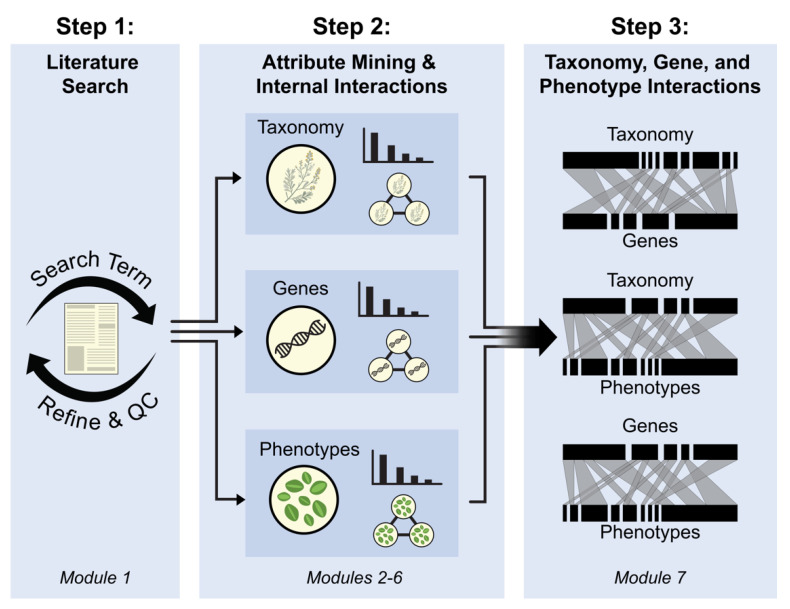
A flowchart showing the progression of a G2PmineR analysis. Abbreviation: QC = Quality Check. See text for more details.

G2PmineR searches abstracts provided to it by the user for sets of species names (abbreviated Ta), gene names (abbreviated G), and phenotype words (abbreviated P) from pre-compiled reference data within the package and allows the user to visualize intersections between the mined results (terms) both within and between sets (Figure 1).

A G2PmineR analysis has three steps and seven modules. The first step has only one module: conducting a literature search and assessing its efficiency (module 1). The second step has five modules (Figure 1): mining Ta (module 2); mining G (module 3); mining P (module 4); summarizing and inferring the consensus of Ta, G and P data (module 5); and conducting internal network analyses for Ta, G and P data (module 6). The third step has a single module, inferring bipartite graphs to link the three datasets (Ta, G, P; module 7).

G2PMineR uses abstracts from a given literature search for all downstream mining applications. Abstracts were used intentionally as they are openly available, and most studied species, genes, and phenotypes of most importance are described in the abstract. We acknowledge that our package is not able to determine if species, genes, or phenotypes are false positives, but after the extensive reading of abstracts and associated text, we determined that false positives due to irrelevant information are rare (98% of 100 randomly sampled abstracts were found to have been mined successfully by our pipeline).

### 2.2. Input Data

The package takes a csv file of abstract text (i.e., one abstract per row, with all text for an abstract between quotes) and a csv file of unique identifiers for each abstract, which can be derived from any scholarly database (e.g., PubMed [21], Scopus [22], or Web of Science [23]). The analysis depends upon included pre-compiled reference data for Ta, G, and P mining, however, the user can also add their own customed reference data (see Section 2.4, Section 2.5, Section 2.6 for more details).

### 2.3. Step 1: Literature Search

#### Module 1: Conduct Literature Search and Assess its Efficiency (Optional)

The user can either perform their literature search in R [19] using easyPubMed, as is done in our vignette, or they can supply the pipeline with abstracts (AbstractStrings in our vignette) and unique identifications (referred to as IDs in the rest of the text) from the scholarly database of their choice (see Section 2.2). In our vignette, we used the EutilsSummary function from the RISmed package [24] to conduct the query and produce IDs. This approach allowed retrieving the PubMed IDs of matching publications and our AbstractsGetteR function was then used to download a vector of abstract text corresponding to the PubMed IDs, outputting AbstractsStrings. AbstractsGetteR relies on the easyPubMed package [25] to perform the retrieval of abstracts.

As an optional first assessment of the efficiency of the literature search, we propose to conduct a preliminary grouping analysis to determine if the abstracts are adequately reflecting the expected search conducted by the user and will therefore be suitable for G2P research: i.e., the fewer groups there are, the more they share in common and therefore the more likely it is that the topics they discuss are relevant to what is being studied. If the user has several groups, they may need to refine their search terms such as adding more precise words (e.g., substituting “monocot” for the more general “plant”). To achieve this, we used two functions here: AbstractsClusterMakeR and MembershipInvestigatoR. AbstractsClusterMakeR defines clusters of abstracts using text2vec [26], qgraph [27], and igraph [28] R packages. We recommend only taking a random sample of AbstractsStrings and IDs (<1000) to run this optional analysis, as it requires a large amount of random access memory (RAM) to run (therefore we do not recommend running this function on a laptop). To delineate a group of abstracts, we used the relaxed word movers’ distance algorithm to calculate similarity scores [26] between abstracts (comparing their whole text) set with a cluster walktrap analysis, which was set at four steps to delimit groups [28]. MembershipInvestigatoR investigates the membership of each group of abstracts by looking at the non-stopwords, which are shared between abstracts. The main output of this function is an object called meminv, a data frame whose columns are “Group” (i.e., group number), “NumberNonStopWords” (i.e., number of non-stopwords shared), “NumberNonStopWordsOverThreshold” (i.e., number of non-stopwords shared over a proportion of abstracts over the user threshold), “WordsOverThreshold” (i.e., non-stopwords shared over a proportion of abstracts over the user threshold, comma separated), “WordsOverThresholdAbstractCounts” (i.e., number of abstracts sharing non-stopwords over the user threshold), and “NumberWordsUnderThreshold” (i.e., number of non-stopwords shared over a proportion of abstracts under the user threshold).

### 2.4. Step 2 Attribute Mining and Internal Interactions

#### 2.4.1. Module 2: Mining Taxonomy (Ta)

In order to provide context to the G2P interactions studied, we mine the abstracts for taxonomy (Ta) to infer which organisms the authors were investigating (Figure 1). This module relies on one function: SpeciesLookeR. This function takes the abstracts strings (AbstractsStrings), unique IDs (IDs), the kingdom of interest (Kingdom, either “P” for Plantae, “A” for Animalia, or “F” for Fungi), and pre-compiled data that are provided with the package based on species from the Global Biodiversity Information Facility (GBIF) [29] that were taxonomically curated using taxize [30] and Taxonstand [31] (this which varies according to the kingdom the user chooses). The user can also decide to add any of their own data as a supplement to the pre-compiled data, in the form of a one-column data frame containing the taxa they wish to add (e.g., “Genus species”) using the flag Add in our package documentation. This function outputs a data frame called AbstractsSpp whose columns are “Genus”, “Species”, and “Matches” (i.e., IDs of abstracts containing species, comma separated). A list of species abbreviations for each taxon found is created by passing AbstractsSpp through SpeciesAbbreviatoR. This module produces a vector of species abbreviations (SppAbbr, e.g., At for *Arabidopsis thaliana*), which is used in the quality-control steps within the GeneLookeR function.

There are some taxa that have names matching English words. These latter taxa are generally from the genera *Cotyledon*, *Codon*, and *Unigenes* for plants, though it could be *Data* or others for animals. The user can also perform manual taxonomical curation on their results if they wish to ensure that only accepted nomenclature is used. If the user desires to do this, they can take the species column of the AbstractsSpp object and pass it through their taxonomical curator of choice.

#### 2.4.2. Module 3: Mining Genes (G)

We mine the abstracts for a set of genes (G) to infer which genes the author is investigating (Figure 1). This module uses two functions: GenesLookeR to perform the mining itself and SynonymReplaceR to harmonize results based on gene nomenclature (since our mining analysis also searches for synonymous names). GenesLookeR takes the arguments abstracts strings (AbstractsStrings), unique IDs (IDs), the kingdom of interest (Kingdom, either “P” for Plantae, “A” for Animalia, or “F” for Fungi), abbreviated species names (SppAbbr), and pre-compiled data that are provided with the package containing the names, families, and ontologies for all of the SwissProt genes for the kingdom of interest as of August 2020 [32]. As in module 2, the user can also decide to add any of their own data as a supplement to the pre-compiled data. In this case, the user has to provide a data frame with three columns: “gene name”, “gene family”, and “gene ontology” (this user-supplied object is called Add in our package documentation). The function outputs a matrix (called GenesOut in our vignette) whose columns are “Gene”, “InOrNot” (i.e., Boolean in at least one abstract or not), “Matches” (i.e., IDs of abstracts containing gene, comma separated), “InSitus” (i.e., exact matches in abstract text), “Family” (i.e., gene family from SwissProt [32]), and “Ontology” (i.e., ontologies from SwissProt [32], comma separated). Note that the “InSitus” column contains original matches and thus may be different to the gene name associated to it (found in the “Gene” column). The functionSynonymReplaceR function takes as arguments GenesOut and Kingdom and replaces occurrences in the “Gene” column with the accepted gene names and combines their outputs if the accepted name was found elsewhere. We chose to restrict our pre-compiled G data to SwissProt curated genes because they are associated with a known ontology and are correctly spelled [32]. After the synonyms are replaced, the user restricts GenesOut so that it only includes abstracts that contain gene term matches as is shown in our vignette. The object created by this restriction is named Genez in our vignette. It has the same column names as GenesOut.

There are two optional functions the user can employ as part of this module: GeneNamesGroupeR creates artificial groups based on numerically stripped gene names, and GeneFrequencySifteR, which excluded genes with a frequency below the threshold chosen by the user. Information about their inputs and outputs can be found in the vignette and manual.

#### 2.4.3. Module 4: Mining Phenotypes (P)

We mine abstracts to infer the phenotypes (P) investigated by the authors (Figure 1). This module is using one function: PhenotypeLookeR. This function takes the abstracts strings (AbstractsStrings), unique IDs (IDs), the kingdom of interest (Kingdom, either “P” for Plantae, “A” for Animalia, or “F” for Fungi), and pre-compiled data that are provided with the package and that vary according to the kingdom chosen. The plant phenotypic words are a manually curated and expanded library of phenotypic words derived primarily from the glossary from the Missouri Botanical Garden website [33]. The animal phenotypic words were derived from the University of California Museum of Palaeontology’s glossary of zoological terms [34]. Finally, the fungi phenotypic words were derived from the University of Adelaide’s glossary of mycological terms [35]. PhenotypeLookeR outputs a data frame called AbsPhen in our vignette whose columns are “PhenoWord” (i.e., phenotypic words), “NumberAbs” (i.e., number of abstracts in which that phenotypic word appeared at least one), “1stWordPair” (most common bigram (i.e., two-word combination) containing this phenotypic word), “2ndWordPair” (second most common bigram containing this phenotypic word), “3rdWordPair” (third most common bigram containing this phenotypic word), and finally “AbsMatches” (i.e., IDs of abstracts containing phenotypic word, comma separated). Considering the first, second, and third most-common bigrams is important to determine the directionality/variety of the phenotypes mined (e.g., root growth vs. root death).

#### 2.4.4. Module 5: Summarizing and Inferring Consensus of Genes, Taxonomy, and Phenotypes Data

We calculate the proportion of abstract matches to see whether the proportion of abstracts that have at least one species, gene, and/or phenotype match is suitable to the user (Figure 1). We use the function AbstractsProportionCalculator to calculate this proportion for each of the mined datasets (Ta, G, P). This function takes as its first argument one of the mining results outputs (AbstractSpp, GenesOut, or AbsPhen). The overall output is a proportion ranging from 0 to 1, representing the proportion of abstracts that have at least one match for this set. Then, the user uses the functions MakeAbstractsSppLongform, MakeGenesOutLongform, and MakeAbsPhenLongform to modify the mined datasets to fit their consensus analysis (for instance, the user can make Ta, G, and/or P datasets that contain only abstracts with at least one match from two or all of the datasets). These functions take their eponymous objects as their single argument (i.e., AbstractsSpp, GenesOut, and AbsPhen, respectively) and they output the long-form versions of them (i.e., versions without concatenating the information; AbstractsSppLong, GenesOutLong, and AbsPhenLong, respectively; see vignette and documentation for more details). The consensus analysis is done using the ConsensusInferreR function. This function takes in the longform datasets inferred in module 5 as well as the native datasets inferred in modules 2, 3, and 4. The user sets whether just two or all of the matches must be present in the finalized consensus data through the arguments of the consensus function itself. For example, if the user wanted to include abstracts that have Ta and G matches but they do not want to include P matches, then they would include Ta = AbstractsSppLong, G = GenesOutLong, P = NULL, AbstractsSpp = AbstractsSpp, GenesOut = GenesOut, and AbsPhen = NULL. The function returns a list where the first object is a data frame with a side-by-side consensus of the inputs (ConsensusMatrix, with the columns “Matches” (the unique IDs of the consensus), and the following two or three columns being the matches of the two or three sets to which the consensus data are restricted), the second object is a Venn diagram showing the abstract-wise intersection of the input datasets built using the package VennDiagram [36], their third through fourth or fifth objects are the original short form matrices but these are restricted to only abstracts meeting the consensus criteria (we called them TaxoCon, GenesCon, and PhenoCon in our vignette, they have the same column names as their original counterparts), and the last object is a vector of abstract IDs in the consensus intersection (ConsensusIDs). The user can either use the raw data or the consensus data for the rest of the analysis. In our vignette we used the raw data.

#### 2.4.5. Module 6: Internal Network Analyses for Ta, G, and P Data

We visualize matching terms using bar graphs to unveil the most heavily used terms within the mined datasets. We use one function here: MatchesBarPlotteR. This function takes the terms (e.g., AbstractsSpp$Species), matching IDs (e.g., AbstractsSpp$Matches), and *n* which denote the number of matches to show a decreasing order of occurrence (we recommend *n* = 25 for visual clarity). The function returns a data frame whose first column is in the top 25 most common terms and the second column is the number of unique abstracts to which they are matched. Base R—or the user’s graphical package of choice—is then used to produce a barplot graph using this data frame as an input.

We visualize internal set results to see the common co-occurrence patterns of terms within sets, i.e., which Ta terms are studied together, which G terms are studied together, and which P terms are studied together (Figure 1). We use two functions here: InternalPairwiseDistanceInferreR and TopN_PickeR_Internal. InternalPairwiseDistanceInferreR takes a vector of terms (e.g., AbstractsSpp$Species) and a vector of matches (e.g., AbstractsSpp$Matches) of the results of one type of mining dataset and infers a pairwise distance matrix between each term using the number of shared ID matches. TopN_PickeR_Internal takes the output of InternalPairwiseDistanceInferreR and subsets it to include only the most similar n (as defined by the user) pairs. The user can use the qgraph function from the qgraph package [27] to create internal relations networks to visualize the results (Figure 1). The overall output are matrices and networks for Ta, G and P.

### 2.5. Step 3: Linking Ta, G, and P Interactions

#### Module 7: Constructing Bipartite Graphs

We integrate the results of the genes, species, and phenotypes analyses using the co-occurrences of these terms so that gene–phenotype, gene–species, and species–phenotype interactions can be visualized in the form of bipartite graphs and accessed more manually through matrices (Figure 1). This module relies on two functions: PairwiseDistanceInferreR, and TopN_PickeR. PairwiseDistanceInferreR takes the terms (e.g., AbstractsSpp$Species and Genez$Gene) and matches (e.g., AbstractsSpp$Matches and Genez$Matches) of the results of two mined datasets and infers a pairwise distance matrix between each term using the number of shared ID matches (e.g., Ta2G, G2P, P2Ta). TopN_PickeR takes the output of PairwiseDistanceInferreR (distance matrix objects that in our vignette are named either PhenoGenes for G2P, GeneSpecies for Ta2G, or PhenoSpecies for P2Ta) and subsets it to include only the most similar number of pairs (*n*), as defined by the user. The user can then use the plotweb function from the bipartite package [37] to create bipartite graphs to visualize the results. The overall output is a matrix whose row names and column names reflect the inputs, which can be used to make the bipartite graphs (Figure 1).

### 2.6. Operating System and R Versioning

G2PmineR has been tested on a MacBook Pro (Cupertino, CA, USA) running MacOS 10.16 and 11.1 using R version 4.0.3 “Bunny-Wunnies Freak Out” [19] in December 2020. It has also been tested on Linux (Ubuntu 18.04.5 LTS; R version 3.6.3 “Holding the Windsock”) (Dell Precision 7920, Round Rock, TX, USA), and Windows 10 (R version 4.0.2 “Taking Off Again”) (Dell Latitude 5501, Round Rock, TX, USA).

## 3. Results

We applied this tool to conduct a literature review of drought tolerance in plants using a subset of 1000 abstracts from the several thousand in PubMed [21] resulting from the search “plant AND drought AND tolerance AND gene”. Overall, the analysis took roughly 3 h to run. The full complement of code, results, and graphs can be found on our website (https://buerkilabteam.github.io/G2PMineR_Web/ (accessed on 2 February 2021)).

### 3.1. Step 1: Literature Search Results

#### Module 1: Conduct Literature Search and Assess its Efficiency Results

The preliminary clustering analysis revealed two unequally sized groups of words shared between >50% of abstracts, indicating potential refinement required for the initial search query (see Table 1). An evaluation of these word groupings revealed that 90% of the words in the smaller group nested within those of the larger group. Because the word groups were similar, we determined that the initial search terms used were sufficient and could continue on with the analysis.

### 3.2. Step 2 Attribute Mining and Internal Interactions Results

#### 3.2.1. Modules 2–4: Mining Results

The taxonomy mining revealed that the abstracts discussed 207 unique taxa as per our database of species and their synonyms, representing 115 genera. Among the abstracts, 64.5% were found to mention at least one plant species. Gene mining revealed that the abstracts discussed 606 unique genes, representing 417 unique gene families. Among the abstracts, 64.4% were found to mention at least one gene in the pre-compiled gene database. Phenotype mining revealed that the abstracts discussed 392 unique phenotype words in our plant-specific database. Among the abstracts, 99.6% mentioned at least one phenotype-associated word.

#### 3.2.2. Module 5: Summarizing and Inferring Consensus of Ta, G, and P Term Matches Results

**Figure 2:**
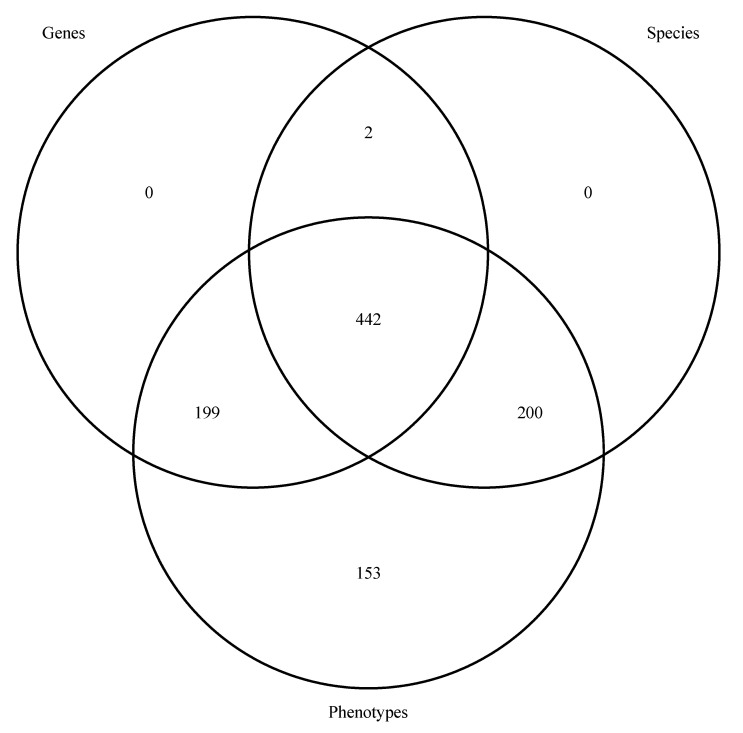
A Venn diagram showing the results of the consensus analysis (module 5) conducted on a subset of abstracts related to drought tolerance in plants. Abbreviations: G = genes; Ta = taxonomy; P = phenotypes.

Less than half (44.3%) of the abstracts had at least one match for all three data types (Ta, G, and P; Figure 2). Among the abstracts, 19.9% had at least one G and P match, but no Ta match. Two hundred (20.0%) abstracts had at least one P and Ta match, but no G match. Only two (0.2%) abstracts had at least one Ta and G match, but no P match. while all abstracts that had at least one G or Ta match matched at least one P as well, 153 (15.3%) abstracts had only a Ta match.

#### 3.2.3. Module 6: Internal Network Analyses for G, Ta and P Data Results

**Figure 3:**
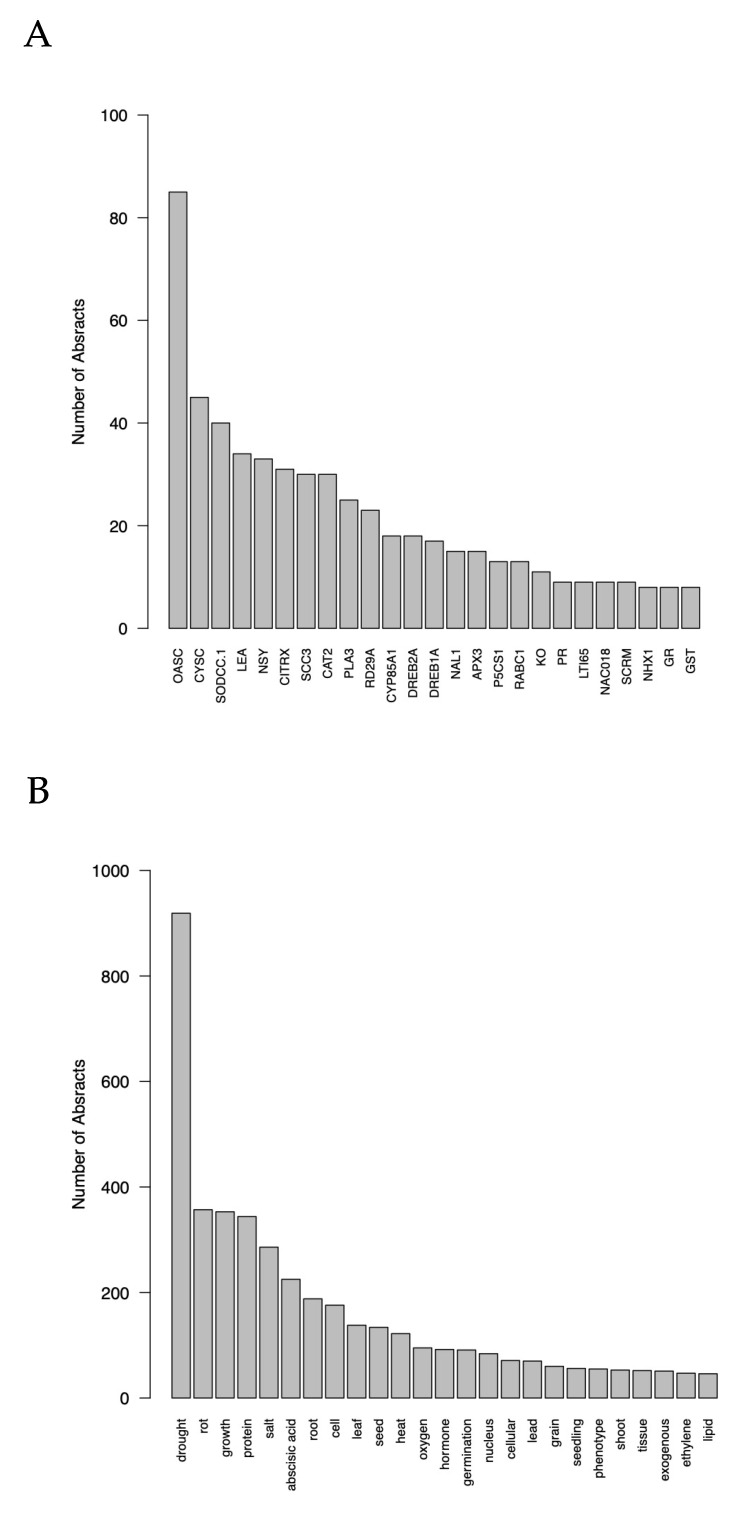
(**A**) Bar plot showing the top 25 genes involved in drought tolerance in plants based on our subset of abstracts inferred using GenesLookeR and MatchesBarPlotteR. (**B**) Bar plot showing the top 25 phenotypes involved in drought tolerance in plants based on our subset of abstracts inferred using PhenotypesLookeR and MatchesBarPlotteR.

The results of the quantitative analysis show that the top five species studied were *Arabidopsis* (spp.), *A. thaliana*, *Oryza sativa*, *Triticum aestivum*, and *Nicotiana tabacum*. The top five genes studied were *OASC*, *CYSC*, *SODCC.1*, *LEA*, and *NSY* (see website for more details). The top five phenotype words studied were “drought”, “rot”, “growth”, “protein”, and “salt” (Figure 3).

**Figure 4:**
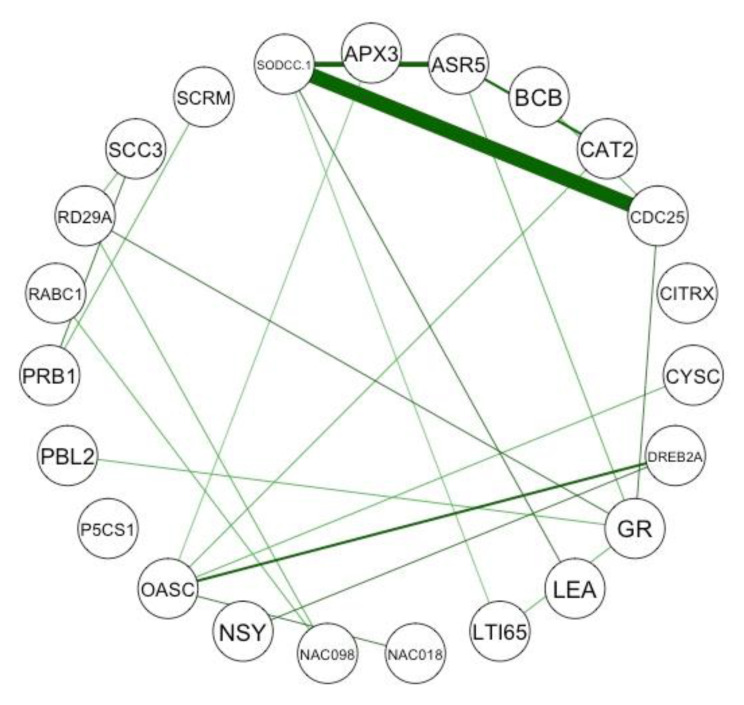
An internal relations networks showing the genes by number of abstracts with at least one occurrence of them shared with other species over a 50% threshold. Genes that appear together in abstracts more often have a wider and more deeply colored bar linking them. Internal relations networks were also produced for the taxonomical and phenotype results but are not shown here. This figure was produced by the functions InternalPairwiseDistanceInferreR and TopN_PickeR_Internal functions from G2PMineR and the qgraph function from qgraph.

The results of the internal interrelations network analysis showed that among taxa, *A. thaliana* often occurred with *T. aestivum*, and that *A. thaliana* also occurred somewhat commonly with *Gossypium hirsutum* and *N. tabacum*. There was also a strong connection between *Sorgum* spp. and *Phyllostachys edulis*. The strongest gene connection (co-occurrence) was between *SODCC.1* and *CDC25* (Figure 4). The second strongest was between *SODCC.1* and *ASR5*, while the third strongest was between *OASC* and *DREB2A* (Figure 4). The phenotype word “drought” was connected to many other words, but most strongly to “salt”, “rot”, “cell”, “root”, “protein”, “growth”, “leaf”, and “abscisic acid” (see our website for more details). There was also a strong connection between “rot” and “protein”, with moderate connections to “salt”, and “growth”.

### 3.3. Step 3: Linking Ta, G, and P Interactions Results

#### Module 7: Constructing Bipartite Graphs Results

**Figure 5:**
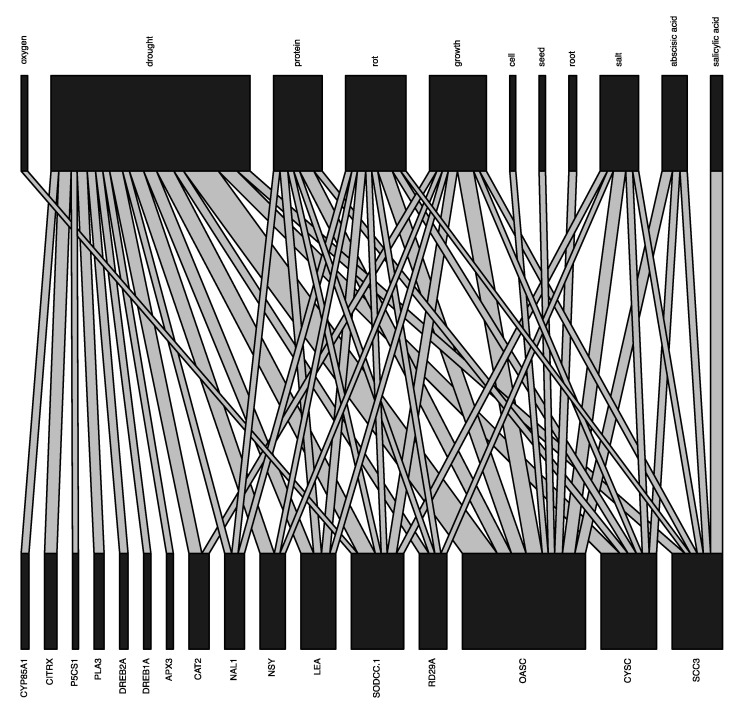
Genes and phenotypes involved in drought tolerance in plants based on our subset of abstracts inferred using the functions PairwiseDistanceInferreR and TopN_PickeR functions from G2PMineR and the plotweb function from bipartite.

The results of the G2P bipartite graph (Figure 5) indicated that *OASC* is heavily associated with the phenotype words “drought”, “protein”, “rot”, “growth”, “salt”, and “abscisic acid” (Figure 5). The phenotype word with the most genes connected to it was “drought”, followed by “rot”, “growth”, “protein”, and “salt” (Figure 5). Some phenotype words were only associated with a single gene in the graph, such *SODCC.1* with “oxygen”, *SCC3* with “salicylic acid”, and “cell”, seed”, and “root” with *OASC* (Figure 5).

Regarding the G2Ta connections, the taxa *Arabidopsis* spp. and *A. thaliana* were connected to all of the genes shown (see our website for more details). *O. sativa* is a very distant third and *Capsicum annuum*, *T. aestivum*, and *Chrysanthemum* spp. tied for fourth. *OASC*, *CYSC*, *RD29A*, and *SODCC.1* were connected to the most species in our top 50 interconnections-restricted distance matrix.

Regarding the P2Ta connections, the taxa *Arabidopsis* sp. and *A. thaliana* were connected to all of the phenotypes shown, and “drought” was the only phenotype word associated to all of the species shown. There was considerable overlap in species studies between “drought”, “salt”, “protein”, “rot”, and “growth”.

## 4. Discussion

### 4.1. Our G2P Analysis Produced Results Aligned with Current Knowledge on Plants Drought Tolerance

Overall, we demonstrated how G2PMiner can perform a high-throughput review of abstracts to gain an unbiased understanding of G2P interactions in a reasonable amount of time on an average laptop (see Section 4.4 for more details). Furthermore, our application of this research workflow to drought tolerance in plants provided G2P results (Figure 5) that corroborated our current understanding of G2P interactions in this field [38,39,40,41,42] indicating to us that this workflow is also effective. For example, *OASC*, one of the genes we found to be most commonly associated to drought stress, codes for cysteine synthase, an enzyme whose transcription is known to be downregulated in drought-stressed plants [41]. On the other hand, *SODCC.1* is known to be upregulated in drought-stressed plants [42]. The product of *SODCC.1* is superoxide dismutase, an enzyme known to neutralize reactive oxygen species, explaining the connection of that gene to oxygen as well as drought [42]. Many of the other genes found in our analysis also make sense within the known G2P drought framework in plants. The taxonomical framework which our analysis unveiled also makes sense as most of the species that co-occurred with drought genes include several models and crop organisms (e.g., *A. thaliana*, *C. annuum*, *T. aestivum*, or *O. sativa*) that also often co-occur with one another. This is logical given that much of our current knowledge of plant–drought interactions is based upon highly studied organisms of human interest such as crop species. We are currently working on a review of genes underpinning drought tolerance in plants based on this package.

### 4.2. G2PMineR Is Applicable to Studying G2P in Plants, Animals, and Fungi

While our example used here delves into the plant kingdom, it is important to note that the package can conduct analyses on data for three kingdoms (Plantae, Animalia, and Fungi), and is therefore useful for a broad range of users. For example, it could be used to investigate drought tolerance in plants, heat tolerance in salmon, and heavy-metal tolerance in fungi.

### 4.3. From Literature Review to Hypothesis Testing

Results of G2PMineR analyses can be used in several ways by scientists at any point in their learning journey. For example, scientists conducting G2P research can use the hypotheses generated by the G2PMineR analysis to mine annotated genomes of their study organisms, or validate their ongoing functional genomic studies using GWAS. On the other hand, undergraduate or graduate students could use the G2PMineR analysis as a backbone for a foundational literature review guiding their thesis research.

The bar plots produced in module six (Figure 3) provide the user with broad context as to the relative abundance of species/genes/phenotypes in the pool of abstracts that they mined. This allows researchers to ascertain whether certain species, genes, or phenotypes may be over-represented in their literature search. The networks produced in module six provide deeper context and allow for hypotheses to begin to be drawn about intra-category relationships (e.g., taxa, genes, phenotypes). For example, the network showing the internal co-occurrence connections among the genes in the pool of abstracts (Figure 4) allows the user to see what genes are commonly studied together, providing a hypothesis of genes that may be expressed together. This hypothesis can be strengthened if two (or more) genes are highly connected in this network and also show connections to the same phenotype(s) in the gene–phenotype bipartite plot. The network showing the internal co-occurrence connections among the phenotypes in the abstracts shows what phenotypes are most commonly studied together, providing a hypothesis of similar underlying mechanisms. This hypothesis can be strengthened if two phenotypes are highly connected in this network and also show connections to the same gene(s) in the gene–phenotype bipartite plot. Finally, the bipartite graphs produced during module seven (Figure 5) allow the user to make hypotheses about inter-category relationships. For example, the gene–phenotype bipartite graph (Figure 5) allows the user to infer the genes that have the strongest co-occurrence connections with their phenotype(s) of interest. This graph also allows the user to see what genes may be expressed together in response to a single (or several) phenotype(s), an observation that can be corroborated by viewing the gene network produced in module six. On the other hand, the genes–taxa bipartite graph allows the use to see in what taxa genes have been studied and may allow for cladistical gene hypotheses to be made.

While abstracts are a valuable source of information to reveal G2P mechanisms, they can be sometimes misleading. For instance, it could be difficult to distinguish whether the noted co-occurrence of gene names and phenotypic traits imply a functional connection or lack of it. Based on our preliminary study, we confirmed this connection by manually inspecting a subset of publications; however, this remains a potential bias of our approach. We are aiming at implementing an additional approach, which would download full texts from freely available publications. This will allow the confirmation of hypotheses generated by G2PMineR.

### 4.4. G2PMineR Was Designed with Diverse Users in Mind

The package also allows for the user to add their own taxonomic, genetic, or phenotypic reference data. This means that the analysis can be customized by each user, and this can allow for the internal compiled databases to be improved iteratively through user–developer interaction. The user can easily access the reference data implemented in the package to evaluate their adequacy in regard to their research question (see website for more details). If the user feels that a specific species/gene/phenotype ought to be included in one of the built-in reference data for broad use, they can submit their addition for consideration by emailing J.M.A.W.

The function LookeRBringeR will return a vector of the unique IDs of the abstracts associated to a term or vector of terms input by the user based on the results of the “-LookeR” functions. The function CoOccBringeR will return a vector of the unique IDs of the abstracts associated to a pair of terms (or two vectors of paired terms) input by the user based on the results of the PairwiseDistanceInferreR function. The user can then use the unique IDs to look at the abstracts or to investigate whether they can access the full papers.

The user does not necessarily have to use all of the functions in the package during their analysis. For example, if they want to mine a set of abstracts (or any other text) for species, genes, or phenotypes they can use one of the “-LookeR” functions to do that. However, one must keep in mind that when running GenesLookeR it requires SpeciesAbbrvs, which means that SpeciesLookeR must be run beforehand. They could then use the results of that function for their own purposes without completing the rest of the analysis.

All of the functions in the package are built to be acceptable for parallelization. This allows the user to analyze large datasets that may take an inordinate amount of time to process serially. This parallelization capability also opens the possibility of the package being hosted remotely and accessed by the user through a web interface, which is made possible by the licensing of the package under the AGPL v. 3.

## 5. Conclusions

G2PMineR is an openly available, iteratively improvable, and useful tool for literature reviews, hypotheses generation, and the successful linking of G2P for plant, animal, or fungi researchers. It is our aspiration that it will become a standard tool in the literature review of G2P projects.

## Figures and Tables

**Table 1:** A comparison of the words shared by over 50% of the abstracts in each group our module 1 analysis found. Words in italic are the search terms. Words with a star (*) next to them are shared between both groups.

Group 1	Group 2
Drought *	Tolerance *
Tolerance *	Drought *
Gene *	Stress *
Stress *	Plant *
Plant *	Gene *
Expression *	Expression *
Response *	Response *
Under *	Transgenic
Study *	Protein
Analysis	Result
	Abiotic
	Role
	Study *
	Under *
	Acid

## Data Availability

The package is available on GitHub (BuerkiLabTeam/G2PMineR) and the vignette data used in this study are available as a pre-compiled object within the package itself (see package documentation) as well as in our website https://buerkilabteam.github.io/G2PMineR_Web/ (accessed on 2 February 2021).
